# Predictors of adverse pregnancy outcomes in severe preeclampsia: A retrospective observational study

**DOI:** 10.1097/MD.0000000000042258

**Published:** 2025-04-25

**Authors:** Ping Li, Yurong Jiang, Yiping You

**Affiliations:** a Obstetrics Department, The Maternal and Child Health Hospital of Hunan Province, Changsha, Hunan, China.

**Keywords:** cholesterol, gestational age, placental growth factor, preeclampsia, pregnancy outcome

## Abstract

Severe preeclampsia (PE) is associated with adverse pregnancy outcomes. The present study aims to identify risk factors contributing to these outcomes in women diagnosed with severe PE. This retrospective observational study included pregnant women diagnosed with severe PE from January 2023 to December 2023. Adverse pregnancy outcomes defined as hypertension accompanied by any of the following: elevated liver enzymes, low platelet count, disseminated intravascular coagulation, placental abruption, pulmonary edema, cerebral hemorrhage, seizures, or death—and adverse fetal outcomes—defined as iatrogenic delivery, small-for-gestational-age, abnormal umbilical hemodynamics, or death. A total of 351 patients with severe PE were included in the analysis (adverse pregnancy outcome group, n = 184; control group, n = 167). Multivariate logistic regression analysis identified gestational age at delivery (odds ratio [OR] = 0.924; 95% confidence interval [CI]: 0.896–0.953, *P* < .001), twin pregnancy (OR = 5.586; 95% CI: 2.774–11.250, *P* < .001), reduced placental growth factor (PlGF) levels (OR = 0.999; 95% CI: 0.998–1.000, *P* = .011), and elevated total cholesterol levels (OR = 1.562; 95% CI: 1.320–1.849, *P* < .001 were independent risk factors for adverse pregnancy outcomes. The combination of gestational age, PlGF, and total cholesterol demonstrated high predictive accuracy, with an area under the curve of 0.867 (95% CI: 0.826–0.908), 79.35% sensitivity, and 90.42% specificity. This study suggests that lower gestational age, twin pregnancy, reduced PlGF, and elevated total cholesterol may be significant risk factors for adverse pregnancy outcomes in women diagnosed with severe PE.

## 1. Introduction

Severe preeclampsia (PE) is a subtype of hypertensive disorder of pregnancy characterized by elevated blood pressure and significant proteinuria.^[1]^ Severe PE affects 2% to 8% of all pregnancies.^[1–3]^ It is a leading cause of maternal and perinatal morbidity and mortality worldwide, making prompt diagnosis and management essential to prevent severe complications.^[4]^ Severe PE is associated with serious maternal complications, including eclampsia, hemolysis, elevated liver enzymes, low platelet count (HELLP) syndrome, stroke, renal failure, and pulmonary edema.^[5]^ For the fetus, it can lead to intrauterine growth restriction, preterm birth, and stillbirth.^[6]^ Early-onset severe PE is associated with worse outcomes compared with late-onset, highlighting the importance of early intervention.^[7]^ Therefore, identifying key risk factors of adverse pregnancy outcomes in severe PE is critical.

Previous studies have demonstrated that risk factors for severe PE were closely associated with adverse pregnancy outcomes. Li et al^[8]^ identified advanced maternal age, multiple pregnancies, the use of assisted reproductive technology, rural residence, a history of hypertensive disorders of pregnancy, carrying a male fetus, multigravida status, polycystic ovary syndrome, HELLP syndrome, intrahepatic cholestasis of pregnancy, cardiovascular disease, gestational diabetes mellitus (GDM), systemic lupus erythematosus, thyroid disease, and liver disease were significantly associated with adverse outcomes in pregnant women with PE. Similarly, Wu et al^[9]^ analyzed 136 women with early-onset severe PE and identified elevated red blood cell counts, reduced platelet counts, and earlier gestational age at delivery as key risk factors for poor pregnancy outcomes.

Additional studies highlighted other potential markers associated with adverse pregnancy outcomes in severe PE, including S100B protein levels and glucose metabolism indices,^[10]^ third space fluid accumulation and twin pregnancies,^[11]^ umbilical hemodynamics and adiponectin levels,^[12]^ serum insulin growth factor-1 and soluble fms-like tyrosine kinase-1 (sFlt-1) levels,^[13]^ and umbilical hemodynamics and microalbuminuria.^[14]^ However, the generalizability of these findings was limited due to factors such as population heterogeneity, the lack of comprehensive biomarker integration, reliance on non-standardized clinical biomarkers, and insufficient validation across diverse clinical settings. Thus, there remains an urgent need to identify clinically accessible risk factors for adverse pregnancy outcomes in women with severe PE, to improve diagnostic accuracy and patient management in a wider range of clinical scenarios. This retrospective study was designed to explore the risk factors for adverse pregnancy outcomes in women with severe PE.

## 2. Methods

### 2.1. Study design

This retrospective observational study enrolled pregnant women diagnosed with severe PE who delivered at Hunan Provincial Maternal and Child Health Hospital from January 2023 to December 2023. Patient data were retrospectively extracted from hospital medical records. This study was approved by the Ethics Committee of Hunan Provincial Maternal and Child Health Hospital (2021-S052). The requirement for individual consent was waived by the committee.

### 2.2. Inclusion criteria and exclusion criteria

The inclusion criteria were (1) meeting the diagnostic criteria for moderate to severe PE as defined by the “Guidelines for Diagnosis and Treatment of Hypertensive Disorders in Pregnancy (2020 Edition)” by the Pregnancy Hypertension Disease Group of the Obstetrics and Gynecology Branch of the Chinese Medical Association^[15]^ and (2) complete clinical data. Severe preeclampsia was defined as the onset of hypertension after 20 weeks of gestation (systolic blood pressure ≥ 160 mm Hg and/or diastolic blood pressure ≥ 110 mm Hg), accompanied by at least one of the following clinical features: persistent headache, visual disturbances or neurological symptoms, persistent upper abdominal pain, elevated liver enzymes (alanine aminotransferase [ALT] or aspartate aminotransferase [AST] ≥ 70 U/L), renal dysfunction (24-hour urine protein > 2.0 g or creatinine > 106 μmol/L), thrombocytopenia (<100 × 10^9^/L), pulmonary edema, or fetal growth restriction with oligohydramnios, fetal death, or placental abruption.

The exclusion criteria were (1) preexisting severe cardiovascular, liver, or renal dysfunction or (2) other serious conditions before pregnancy. The severe PE patients with adverse maternal or fetal outcomes were included in the adverse pregnancy outcome group, while the women without adverse pregnancy outcomes were included in the control group. No specific gestational age cutoff was applied; all eligible patients diagnosed with severe preeclampsia based on the above diagnostic criteria within the defined study period were included.

### 2.3. Data collection

Baseline characteristics were retrospectively extracted, including maternal age, body mass index, gestational age at delivery, history of gestational hypertension, GDM, hepatitis virus infection, twin pregnancy, use of in vitro fertilization-embryo transfer (IVF-ET), and intensive care unit (ICU) admission. Laboratory parameters collected included placental growth factor (PlGF), total cholesterol, triglycerides, liver enzymes (ALT and AST), and platelet counts. Fetal characteristics recorded included estimated fetal weight, fetal growth status, and umbilical artery Doppler findings. These variables were retrospectively collected from standard clinical assessments conducted at initial hospital admission.

### 2.4. Fetal weight estimation

Fetal weight was estimated using ultrasonography measurements, including biparietal diameter, head circumference (HC), abdominal circumference (AC), and femur length (FL), applying the Hadlock formula: estimated fetal weight = 10^(1.326+0.0107×HC+0.0438×AC+0.158×FL−0.00326×AC×FL)^.

### 2.5. Definition of outcomes

The adverse pregnancy outcomes included adverse maternal outcomes and adverse fetal outcomes. The adverse maternal outcomes were defined as the presence of hypertension (blood pressure ≥ 140/90 mm Hg on 2 occasions 2 hours to 2 weeks apart) plus one of the following: elevated AST or ALT (≥80 U/L), platelet count ≤ 100 × 10^9^/L, disseminated intravascular coagulation, abruption (clinical and/or pathological), pulmonary edema, cerebral hemorrhage, seizure (in a woman without underlying seizure disorder), acute renal failure (creatinine ≥ 114.4 μmol/L), or maternal death.^[16]^ The adverse fetal outcomes included iatrogenic delivery indicated for hypertensive complications of pregnancy as reported by the primary obstetrician, small-for-gestational-age (SGA) birth weight (≤10th percentile for gestational age), abnormal umbilical artery Doppler (absent or reverse flow), fetal death, or neonatal death.^[17]^

### 2.6. Postpartum follow-up

Participants underwent follow-up assessments at 6 and 12 weeks postpartum, including blood pressure monitoring, urine protein measurement, liver and kidney function tests, routine blood and coagulation analyses, and additional cardiac evaluations such as ECG and echocardiography as necessary, to identify and manage adverse symptoms or signs timely.

### 2.7. Statistical analysis

The sample size was determined by including all eligible severe PE cases meeting inclusion criteria within the defined study period. No formal sample size calculation was performed due to the retrospective nature of this observational study. Statistical analysis was performed using SPSS version 19.0 software (IBM Corp., Armonk, NY). For continuous variables, normality tests were conducted, if the data follow a normal distribution, continuous variables are presented as mean ± standard deviation and compared using Student *t* test, while non-normally distributed variables are presented as median (inter-quartile range) and compared using Mann–Whitney *U* test. Categorical variables are expressed as numbers and percentages, and the chi-squared (*χ*^2^) test was used for comparison between groups. Multivariate logistic regression was employed to investigate the factors independently associated with adverse pregnancy outcomes. Variables that were statistically significant in the univariate analyses were included in the multivariate logistic regression model. Receiver operating characteristic (ROC) curves were generated to assess the diagnostic performance of significant predictors identified in the logistic regression analysis. The area under the curve (AUC) was calculated to evaluate the overall predictive accuracy. Two-sided *P* < .05 was considered as statistical significance.

## 3. Results

### 3.1. Baseline information

The study included 351 severe PE patients, 184 of whom experienced adverse pregnancy outcomes. The adverse pregnancy outcome group had a significantly lower gestational age at delivery compared with the control group (*P* < .001). The frequencies of twin pregnancy, use of IVF-ET, and ICU admission were higher in the adverse outcome group (*P* < .05). In addition, the adverse outcome group exhibited significantly lower levels of PlGF and higher levels of triglycerides and total cholesterol compared with the control group (*P* < .05) (Table 1). There were no significant differences between groups regarding highest recorded systolic (*P* = .171) and diastolic (*P* = .863) blood pressure (Table 1), nor the use of antihypertensive medications (magnesium sulfate, nifedipine, labetalol; all *P* > .05, Table S1, Supplemental Digital Content, https://links.lww.com/MD/O753).

**Table 1:** Baseline characteristics of the study population.

Characteristics	Control group (n = 167)	Adverse outcome group (n = 184)	*P*
Age (years)	31.46 ± 4.83	31.89 ± 5.29	.428
BMI (kg/m^2^)	29.18 ± 3.77	28.96 ± 3.90	.594
Weight gain (kg)	14.98 ± 5.19	14.46 ± 5.63	.376
IVF-ET, n (%)	30 (17.96)	55 (29.89)	.009
Twin pregnancy, n (%)	12 (7.19)	52 (28.26)	<.001
HBV, n (%)	9 (5.39)	10 (5.43)	.985
GDM, n (%)	54 (32.34)	55 (29.89)	.621
Hyperlipidemia, n (%)	98 (58.68)	89 (48.37)	.053
ICU admission, n (%)	21 (12.57)	66 (35.87)	<.001
Gestational age (wk)	37.76 ± 1.65	34.43 ± 2.94	<.001
Number of pregnancies	2 (1, 3)	2 (1, 3)	.815
Number of deliveries	0 (0, 1)	0 (0, 1)	.539
PlGF (pg/mL)	205.1 (96.2, 343.9)	91.2 (40.07, 194.78)	<.001
Triglycerides (mmol/L)	3.64 (2.93, 4.92)	4.24 (3.27, 5.43)	.014
Total cholesterol (mmol/L)	6.31 (5.32, 7.09)	6.54 (5.69, 7.58)	.018
Highest systolic BP (mm Hg)	162 (154, 170)	160 (153, 168)	.171
Highest diastolic BP (mm Hg)	102 (95, 110)	103 (97.25, 110)	.863

BMI = body mass index, BP = blood pressure, GDM = gestational diabetes mellitus, HBV = hepatitis B virus, ICU = intensive care unit, IVF-ET = in vitro fertilization-embryo transfer, PlGF = placental growth factor.

### 3.2. Neonatal outcomes

The neonatal characteristics between the 2 groups were compared (Table 2). Neonates in the adverse outcome group had significantly lower birth weight compared to the control group (2038.04 ± 625.37 g vs 2965.57 ± 476.94 g, *P* < .001). Additionally, the adverse outcome group showed significantly higher proportions of SGA infants (47.28% vs 14.97%, *P* < .001), abnormal umbilical artery Doppler findings (22.28% vs 1.80%, *P* < .001), and neonatal intensive care unit admission (59.78% vs 5.39%, *P* < .001) compared to the control group. No significant differences were observed between groups for neonatal death, fetal death (stillbirth), or proportion of live births (all *P* = 1.000).

**Table 2:** Neonatal characteristics of study population.

Neonatal characteristics	Control group (n = 167)	Adverse outcome group (n = 184)	*t*/*χ*²	*P*-value
Birth weight (g)	2965.57 ± 476.94	2038.04 ± 625.37	15.506	<.001
Fetal growth restriction (FGR), n (%)	25 (14.97%)	87 (47.28%)	42.070	<.001
Abnormal umbilical artery Doppler, n (%)	3 (1.80%)	41 (22.28%)	33.510	<.001
Neonatal death, n (%)	0 (0.00%)	0 (0.00%)	0.000	1.000
Stillbirth (fetal death), n (%)	0 (0.00%)	0 (0.00%)	0.000	1.000
Live births, n (%)	167 (100%)	184 (100%)	0.000	1.000
NICU admission, n (%)	9 (5.39%)	110 (59.78%)	115.584	<.001

NICU = neonatal intensive care unit.

### 3.3. Univariate and multivariate logistic regression analysis

Univariate logistic regression analysis identified several significant factors associated with adverse pregnancy outcomes, including lower gestational age at delivery (odds ratio [OR] = 0.494, 95% confidence interval [CI]: 0.422–0.579, *P* < .001), twin pregnancy (OR = 5.088, 95% CI: 2.606–9.936, *P* < .001), IVF-ET use (OR = 1.947, 95% CI: 1.174–3.228, *P* = .010), ICU admission (OR = 3.889, 95% CI: 2.249–6.725, *P* < .001), reduced PlGF (OR = 0.998, 95% CI: 0.997–0.999, *P* = .001), and elevated total cholesterol (OR = 1.210, 95% CI: 1.043–1.403, *P* = .012). Variables including triglycerides, maternal age, body mass index, GDM, and hepatitis B virus infection were not significantly associated with adverse pregnancy outcomes (all *P* > .05) (Table S2, Supplemental Digital Content, https://links.lww.com/MD/O754).

Multivariate logistic regression showed that lower gestational age at delivery (OR = 0.924; 95% CI: 0.896–0.953, *P* < .001), twin pregnancy (OR = 5.586; 95% CI: 2.774–11.250, *P* < .001), reduced PlGF levels (OR = 0.999; 95% CI: 0.998–1.000, *P* = .011), and elevated total cholesterol levels (OR = 1.562; 95% CI: 1.320–1.849, *P* < .001) were independently associated with an increased risk of adverse pregnancy outcomes (Table 3).

**Table 3:** Multivariate logistic regression analysis of factors influencing adverse pregnancy outcomes of severe preeclampsia.

Characteristics	OR	95% CI	*P*
Gestational age	0.924	0.896–0.953	<.001
Twin pregnancy	5.586	2.774–11.250	<.001
PlGF	0.999	0.998–1.000	.011
Total cholesterol	1.562	1.320–1.849	<.001

CI = confidence interval, OR = odds ratio, PlGF = placental growth factor.

**Figure 1.**
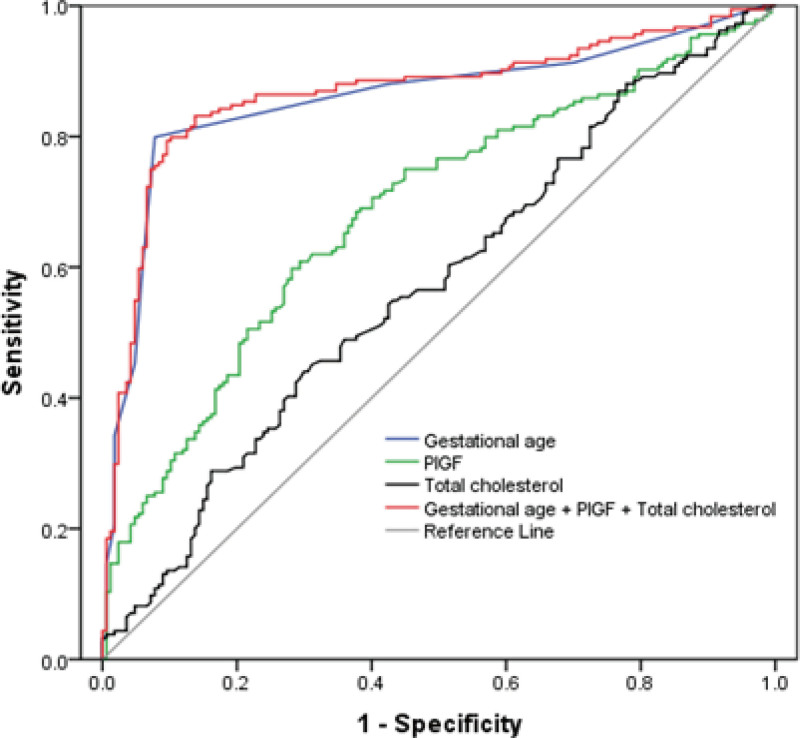
Receiver operating characteristic curves for predicting adverse pregnancy outcomes in severe preeclampsia. The ROC curves display the predictive performance of different clinical and biochemical markers for adverse pregnancy outcomes in women with severe preeclampsia. The blue line represents the gestational age model. The green line represents the placental growth factor (PlGF) model. The black line represents the total cholesterol model. The red line represents the combined model incorporating gestational age, PlGF, and total cholesterol. The diagonal line represents the reference line of no discrimination (area under the curve (AUC) = 0.5). The combined model shows the highest AUC, indicating superior predictive accuracy for identifying high-risk patients. Sensitivity and specificity values are plotted on the *Y* and *X* axes, respectively.

ROC curve analysis was conducted to assess the diagnostic performance of gestational age, PlGF, total cholesterol, and their combination in predicting adverse pregnancy outcomes in severe preeclampsia (Fig. 1). Twin pregnancy was not analyzed in the ROC curve analysis because there are possible factors of human selection in twins. The analysis revealed that the combination of gestational age, PlGF, and total cholesterol had the highest predictive value, with an AUC of 0.867 (95% CI: 0.826–0.908). The sensitivity and specificity of this combined model were 79.35% and 90.42%, respectively, indicating strong predictive accuracy. Gestational age had the highest AUC (0.858; 95% CI: 0.816–0.901), followed by PlGF (AUC: 0.685; 95% CI: 0.629–0.740) and total cholesterol (AUC: 0.573; 95% CI: 0.513–0.633) (Figure S1, Table S3, Supplemental Digital Content, https://links.lww.com/MD/O755.).

## 4. Discussion

This study showed that lower gestational age, twin pregnancy, reduced PlGF levels, and elevated total cholesterol were independently associated with pregnancy outcomes in women with severe PE. These findings underscore the importance of early identification of these risk factors to improve risk stratification and clinical management in this high-risk population.

The findings of this study suggested that gestational age at delivery may be a pivotal risk factor for adverse pregnancy outcomes in severe PE patients. This observation aligned with the well-established evidence that preterm delivery, often necessitated by the progression of severe PE, was a major contributor to neonatal morbidity and mortality.^[18]^ Earlier gestational age at the time of delivery was associated with poorer neonatal outcomes, irrespective of the degree of PE severity.^[19]^ A recent systematic review further emphasized that delaying delivery in cases of multiple pregnancy significantly improved neonatal outcomes by extending gestational age, highlighting the delicate balance clinicians must maintain between prolonging pregnancy to improve fetal maturity and managing escalating maternal risks.^[20]^ Increasing gestational age was also associated with an increasing cumulative risk of adverse maternal outcomes in patients with PE.^[21,22]^ Wu et al^[9]^ showed that an earlier delivery week was a risk factor for adverse pregnancy outcomes in women with severe PE alongside elevated red blood cell count and reduced platelet count. Barda et al similarly highlighted that earlier gestational age at the initial presentation of hypertensive disorders was independently predictive of progression to severe features of preeclampsia, reinforcing gestational age as a critical factor in managing severe PE.^[23]^ Additionally, recent evidence indicates an exponential increase in the incidence and severity of PE with advancing gestational age, underscoring the importance of gestational timing in clinical decision-making.^[24]^ The need for premature intervention due to worsening maternal or fetal conditions often compromises neonatal outcomes, highlighting the delicate balance between prolonging pregnancy and mitigating the escalating risks of severe PE. Our findings reinforce this evidence by confirming that lower gestational age at delivery remains a major independent predictor of adverse pregnancy outcomes.

The study also identified twin pregnancy as a significant risk factor for adverse outcomes, corroborating previous research that multiple gestations increased the risk of hypertensive disorders and pregnancy complications.^[25]^ Multiple pregnancies imposed additional physiological demands, such as increased uteroplacental blood flow and heightened uterine tension, which can exacerbate the risks associated with severe PE.^[26]^ Zhang et al^[11]^ showed that twin pregnancy was associated with adverse maternal outcomes in severe PE. Twin pregnancies increase preeclampsia risk by 2 to 3 times compared with singleton pregnancies, typically resulting in earlier onset, faster progression, and greater severity of PE, increasing the likelihood of end-organ damage, placental abruption, and fetal complications such as growth restriction and premature delivery.^[27]^ Mechanistically, twin pregnancies increase placental mass and circulating antiangiogenic factors such as sFlt-1, resulting in a marked imbalance between angiogenic and antiangiogenic factors, endothelial dysfunction, and exacerbation of maternal inflammatory responses, ultimately intensifying PE severity. Additionally, twin pregnancies are associated with earlier gestational age at delivery and significantly higher incidences of preterm preeclampsia compared to singletons, emphasizing the heightened clinical challenge these pregnancies present.^[28]^ From a clinical perspective, this finding suggests that pregnancies involving multiple fetuses should be closely monitored for signs of hypertensive disorders, with a low threshold for intervention to manage severe PE, potentially improving maternal and fetal outcomes.

Reduced serum PlGF was another key finding in this study, aligning with the role of PlGF as a biomarker for placental health and disease severity. Reduced PlGF levels are significantly associated with severe PE and are associated with a higher incidence of adverse outcomes, such as intrauterine fetal death, placental abruption, HELLP syndrome, and SGA infants.^[29,30]^ A reduction in PlGF disrupted placental and fetal blood flow, contributing to poor fetal growth and increasing the risk of adverse outcomes.^[31]^ Mechanistically, PlGF is essential for normal placental development as it facilitates angiogenesis and proper vascular remodeling. Reduced PlGF levels impair spiral artery remodeling, resulting in inadequate placental perfusion and chronic placental ischemia, a hallmark of preeclampsia. This chronic ischemia further amplifies oxidative stress, inflammatory responses, and endothelial dysfunction, intensifying PE severity.^[32,33]^ Furthermore, reduced PlGF levels during the first trimester significantly precede clinical manifestations, underscoring its potential predictive value for identifying patients at risk of severe PE.^[33,34]^ These findings highlighted the potential usefulness of PlGF levels in clinical practice as a biomarker for identifying patients at higher risk of adverse outcomes, enabling earlier and more targeted interventions. Regular monitoring of PlGF could potentially guide therapeutic strategies to optimize placental function and improve pregnancy outcomes. Recent clinical research has also suggested that therapeutic supplementation or modulation of PlGF levels may represent a novel treatment approach, highlighting its potential clinical significance beyond risk prediction.^[33]^

Identifying elevated total cholesterol levels as a significant predictor was also consistent with existing research on lipid metabolism in pregnancy. Studies have shown that high triglyceride and low-density lipoprotein cholesterol levels during early and late pregnancy were associated with several complications, such as GDM, PE, preterm birth, and intrahepatic cholestasis of pregnancy. In contrast, higher high-density lipoprotein cholesterol levels may offer protective benefits.^[35–38]^ Dyslipidemia contributed to systemic inflammation, oxidative stress, and endothelial dysfunction, all of which played roles in the pathogenesis of PE. Elevated cholesterol levels, particularly increased low-density lipoprotein cholesterol and triglycerides along with reduced high-density lipoprotein cholesterol, are strongly linked with maternal endothelial dysfunction. Increased cholesterol and triglycerides lead to oxidative stress by accumulating in endothelial cells, subsequently impairing endothelial function and reducing prostacyclin release, exacerbating the clinical severity of PE.^[39]^ These metabolic disturbances can exacerbate the clinical manifestations of severe PE, leading to complications such as placental abruption, fetal growth restriction, and preterm delivery.^[40,41]^ A study on Chinese women showed an increased risk of adverse pregnancy outcomes.^[37]^ A recent meta-analysis confirmed that women who develop preeclampsia consistently exhibit elevated levels of total cholesterol and triglycerides, supporting cholesterol as an important biomarker for identifying high-risk pregnancies.^[42]^ Decreasing blood lipid levels could decrease the risk of adverse maternal or fetal outcomes in patients with PE.^[43]^ Clinically, it suggested that managing lipid levels could be crucial in reducing the risk of severe PE and its associated complications.

Our study also demonstrated significant neonatal implications of severe PE, including notably lower birth weight, higher incidence of FGR, increased neonatal intensive care unit admissions, and abnormal umbilical artery Doppler findings. These neonatal complications directly result from impaired placental perfusion and fetal growth restriction, characteristic of severe PE. Consistent with previous studies, these findings underscore the importance of early fetal monitoring and timely interventions to minimize neonatal morbidity associated with severe PE.^[44–46]^ Interestingly, our analysis showed no significant differences in antihypertensive medication usage (magnesium sulfate, nifedipine, and labetalol) between the adverse outcome group and the control group. This finding indicates that the observed differences in maternal and neonatal outcomes are unlikely attributed to disparities in hypertension management between groups, reinforcing the importance of other identified predictors such as gestational age, PlGF levels, and lipid profiles.

The present study identified a combination of gestational age, PlGF, and total cholesterol as an effective predictive model for adverse pregnancy outcomes in severe PE. Rana et al demonstrated that combining angiogenic factors like PlGF and sFlt-1 enhanced the prediction of adverse outcomes over single markers.^[47]^ Including gestational age and cholesterol extends this approach, providing a more comprehensive assessment by integrating clinical and biochemical data. Similarly, Sun et al^[13]^ showed high predictive value with combined serum markers such as insulin growth factor-1 and sFlt-1, while incorporating gestational age in the present model offers additional relevance for clinical decision-making on delivery timing. The study by Rana et al^[48]^ on twin pregnancies also supported the use of combined markers, showing that integrating angiogenic factors with gestational age improves outcome predictions. While Reddy et al^[49]^ noted that adding cardiovascular indices did not outperform angiogenic markers alone, the simpler model proposed here, combining accessible clinical measures with PlGF, achieved comparable predictive strength. Verlohren et al^[50]^ further validated the utility of combining PlGF with other markers for enhanced prediction. The findings supported adopting this integrated model to refine risk assessment and management strategies in severe PE, potentially improving maternal and fetal outcomes. Few studies were available regarding the factors for adverse pregnancy outcomes, specifically in severe PE.^[8–11,13,14]^ Further research is necessary to optimize these predictive models, but this study provides a practical framework based on widely accessible data.

This study has several limitations, including a small sample size, a single-center design, and the potential for biases due to retrospective data collection. These factors may limit the generalizability of the findings and the applicability of the predictive model. Future studies should involve multicenter studies with larger sample sizes to verify the robustness of the findings of this study and further explore additional influencing factors. In addition, laboratory parameters may vary depending on gestational age. Since analyses were performed close to delivery, differences in gestational ages between groups represent a potential limitation. Uterine artery Doppler assessment was not included in this study due to incomplete data availability for many participants. This omission may have limited our ability to fully evaluate the predictive value of Doppler ultrasound indices in this cohort. Future prospective studies with systematically collected uterine artery Doppler data are recommended to improve predictive modeling and clinical decision-making for severe preeclampsia.

## 5. Conclusion

This study indicates that low gestational age, twin pregnancy, reduced PlGF levels, and elevated total cholesterol are potential independent risk factors for adverse pregnancy outcomes in women with severe PE. These findings highlight the need for early identification and monitoring of these risk factors to improve risk assessment and clinical management in this high-risk population.

## Author contributions

**Conceptualization:** Ping Li.

**Writing – original draft:** Ping Li, Yurong Jiang, Yiping You.

**Writing – review & editing:** Ping Li, Yurong Jiang, Yiping You.

## Supplementary Material



## References

[1] Lisonkova S Bone JN Muraca GM. Incidence and risk factors for severe preeclampsia, hemolysis, elevated liver enzymes, and low platelet count syndrome, and eclampsia at preterm and term gestation: a population-based study. Am J Obstet Gynecol. 2021;225:538.e1–538.e19. 10.1016/j.ajog.2021.04.261 33974902

[2] Kongwattanakul K Saksiriwuttho P Chaiyarach S Thepsuthammarat K. Incidence, characteristics, maternal complications, and perinatal outcomes associated with preeclampsia with severe features and HELLP syndrome. Int J Womens Health. 2018;10:371–7. 30046254 10.2147/IJWH.S168569 PMC6054275

[3] Ngwenya S. Severe preeclampsia and eclampsia: incidence, complications, and perinatal outcomes at a low-resource setting, Mpilo Central Hospital, Bulawayo, Zimbabwe. Int J Womens Health. 2017;9:353–7. 28553148 10.2147/IJWH.S131934 PMC5439934

[4] Ye C Ruan Y Zou L. The 2011 survey on hypertensive disorders of pregnancy (HDP) in China: prevalence, risk factors, complications, pregnancy and perinatal outcomes. PLoS One. 2014;9:e100180. 24937406 10.1371/journal.pone.0100180 PMC4061123

[5] Hansson SR Nääv A Erlandsson L. Oxidative stress in preeclampsia and the role of free fetal hemoglobin. Front Physiol. 2014;5:516. 25628568 10.3389/fphys.2014.00516 PMC4292435

[6] Backes CH Markham K Moorehead P Cordero L Nankervis CA Giannone PJ. Maternal preeclampsia and neonatal outcomes. J Pregnancy. 2011;2011:214365. 21547086 10.1155/2011/214365 PMC3087144

[7] Teka H Yemane A Abraha HE. Clinical presentation, maternal-fetal, and neonatal outcomes of early-onset versus late onset preeclampsia–eclampsia syndrome in a teaching hospital in a low-resource setting: a retrospective cohort study. PLoS One. 2023;18:e0281952. 36848332 10.1371/journal.pone.0281952 PMC9970097

[8] Li X Zhang W Lin J. Risk factors for adverse maternal and perinatal outcomes in women with preeclampsia: analysis of 1396 cases. J Clin Hypertens (Greenwich). 2018;20:1049–57. 29707880 10.1111/jch.13302 PMC8031100

[9] Wu SW Wu LF Wang Q Zhang WY. Risk factors of adverse pregnancy outcomes during expectant management of early onset severe pre-eclampsia. Chin J Obstet Gynecol. 2010;45:165–9. 20450750

[10] Yan A. Application value of serum S100B combined with glucose metabolism indexes in predicting adverse pregnancy outcomes of patients with severe preeclampsia. J Hum Hypertens. 2024;38:232–7. 38160207 10.1038/s41371-023-00887-x

[11] Zhang LN Wang ZZ Wu JL. Effect of third interstitial fluid on adverse outcomes in patients with severe pre-eclampsia and twin pregnancy: a 5-year single-center retrospective study. Curr Med Sci. 2023;43:1213–20. 38079055 10.1007/s11596-023-2815-5

[12] Zhang Z Liu F Zhang Q Li D Cai L. Umbilical artery ultrasound haemodynamics combined with serum adiponectin levels can aid in predicting adverse pregnancy outcomes in patients with severe pre-eclampsia. J Obstet Gynaecol. 2023;43:2232656. 37462393 10.1080/01443615.2023.2232656

[13] Sun Y Meng C Yu L. Correlation of serum IGF-1 and sFlt-1 levels with adverse pregnancy outcomes in patients with severe preeclampsia. Altern Ther Health Med. 2023;29:364–9. 37171953

[14] Luo Y Li Y Zhang L. The combined use of ultrasound examination of hemodynamics in the umbilical artery and urine microalbumin levels can predict adverse pregnancy outcomes in patients with severe preeclampsia. J Obstet Gynaecol. 2023;43:2208674. 37227086 10.1080/01443615.2023.2208674

[15] Hypertensive Disorders in Pregnancy Group, Chinese Society of Obstetrics and Gynecology. Guidelines for the diagnosis and treatment of hypertensive disorders in pregnancy (2020). Chin J Obst Gynecol. 2020;55:227–38.

[16] Li P Jiang Y You Y. Serum placental growth factor, total cholesterol, and triglycerides for prediction of intrahepatic cholestasis of pregnancy. Medicine (Baltim). 2023;102:e36178. 10.1097/MD.0000000000036178 PMC10727609 38115361

[17] Roberts JM Myatt L Spong CY. Vitamins C and E to prevent complications of pregnancy-associated hypertension. N Engl J Med. 2010;362:1282–91. 20375405 10.1056/NEJMoa0908056 PMC3039216

[18] Iacobelli S Bonsante F Robillard PY. Comparison of risk factors and perinatal outcomes in early onset and late onset preeclampsia: a cohort based study in Reunion Island. J Reprod Immunol. 2017;123:12–6. 28858635 10.1016/j.jri.2017.08.005

[19] Hauptman I Gill KS Lim T Mack WJ Wilson ML. Prediction of neonatal outcomes using gestational age vs ACOG definitions of maternal disease severity in hypertensive disorders of pregnancy. Arch Gynecol Obstet. 2025;311:639–48. 39152282 10.1007/s00404-024-07684-y PMC11920366

[20] Cheung KW Seto MTY Wang W Lai CWS Kilby MD Ng EHY. Effect of delayed interval delivery of remaining fetus(es) in multiple pregnancies on survival: a systematic review and meta-analysis. Am J Obstet Gynecol. 2020;222:306–19.e18. 31394069 10.1016/j.ajog.2019.07.046

[21] Pettit F Mangos G Davis G Henry A Brown MA. Pre-eclampsia causes adverse maternal outcomes across the gestational spectrum. Pregnancy Hypertens. 2015;5:198–204. 25943645 10.1016/j.preghy.2015.02.002

[22] Zheng J Zhang L Zhou Y Xu L Zhang Z Luo Y. Development and evaluation of a nomogram for adverse outcomes of preeclampsia in Chinese pregnant women. BMC Pregnancy Childbirth. 2022;22:504. 35725446 10.1186/s12884-022-04820-x PMC9210655

[23] Barda S Yoeli Y Stav N. Factors associated with progression to preeclampsia with severe features in pregnancies complicated by mild hypertensive disorders. J Clin Med. 2023;12:7022. 38002636 10.3390/jcm12227022 PMC10672209

[24] Ohkuchi A Suzuki H Matsubara K. Exponential increase of the gestational-age-specific incidence of preeclampsia onset (COPE study): a multicenter retrospective cohort study in women with maternal check-ups at <20 weeks of gestation in Japan. Hypertens Res. 2022;45:1679–89. 36109601 10.1038/s41440-022-01013-z

[25] Nawsherwan Liu Z Le Z. The adverse effect of gestational diabetes mellitus and hypertensive disorders of pregnancy on maternal–perinatal outcomes among singleton and twin pregnancies: a retrospective cohort study (2011–2019). Front Endocrinol (Lausanne). 2023;14:1267338. 38098860 10.3389/fendo.2023.1267338 PMC10720659

[26] Krotz S Fajardo J Ghandi S Patel A Keith LG. Hypertensive disease in twin pregnancies: a review. Twin Res. 2002;5:8–14. 11893276

[27] Wang Y Wu N Shen H. A review of research progress of pregnancy with twins with preeclampsia. Risk Manag Healthc Policy. 2021;14:1999–2010. 34040463 10.2147/RMHP.S304040 PMC8140947

[28] Francisco C Gamito M Reddy M Rolnik DL. Screening for preeclampsia in twin pregnancies. Best Pract Res Clin Obstet Gynaecol. 2022;84:55–65. 35450774 10.1016/j.bpobgyn.2022.03.008

[29] Turpin CA Sakyi SA Owiredu WK Ephraim RK Anto EO. Association between adverse pregnancy outcome and imbalance in angiogenic regulators and oxidative stress biomarkers in gestational hypertension and preeclampsia. BMC Pregnancy Childbirth. 2015;15:189. 26303772 10.1186/s12884-015-0624-y PMC4549075

[30] Lecarpentier E Gris JC Cochery-Nouvellon E. Urinary placental growth factor for prediction of placental adverse outcomes in high-risk pregnancies. Obstet Gynecol. 2019;134:1326–32. 31764746 10.1097/AOG.0000000000003547

[31] Diguisto C Piver E Gouge AL. First trimester uterine artery Doppler, sFlt-1 and PlGF to predict preeclampsia in a high-risk population. J Matern Fetal Neonatal Med. 2017;30:1514–9. 27151901 10.1080/14767058.2016.1183631

[32] Creswell L O’Gorman N Palmer KR da Silva Costa F Rolnik DL. Perspectives on the use of placental growth factor (PlGF) in the prediction and diagnosis of pre-eclampsia: Recent insights and future steps. Int J Womens Health. 2023;15:255–71. 36816456 10.2147/IJWH.S368454 PMC9936876

[33] Chau K Hennessy A Makris A. Placental growth factor and pre-eclampsia. J Hum Hypertens. 2017;31:782–6. 29115294 10.1038/jhh.2017.61 PMC5680413

[34] Velegrakis A Kouvidi E Fragkiadaki P Sifakis S. Predictive value of the sFlt‑1/PlGF ratio in women with suspected preeclampsia: an update (review). Int J Mol Med. 2023;52:89. 37594116 10.3892/ijmm.2023.5292 PMC10500221

[35] Wang C Zhu W Wei Y. The associations between early pregnancy lipid profiles and pregnancy outcomes. J Perinatol. 2017;37:127–33. 27787507 10.1038/jp.2016.191

[36] Rogne T Gill D Liew Z. Mediating factors in the association of maternal educational level with pregnancy outcomes: a mendelian randomization study. JAMA Netw Open. 2024;7:e2351166. 38206626 10.1001/jamanetworkopen.2023.51166 PMC10784860

[37] Jin WY Lin SL Hou RL. Associations between maternal lipid profile and pregnancy complications and perinatal outcomes: a population-based study from China. BMC Pregnancy Childbirth. 2016;16:60. 27000102 10.1186/s12884-016-0852-9 PMC4802610

[38] Asltoghiri M Moghaddam-Banaem L Behboudi-Gandevani S Rahimi Froushani A Ramezani Tehrani F. Prediction of adverse pregnancy outcomes by first-trimester components of metabolic syndrome: a prospective longitudinal study. Arch Gynecol Obstet. 2023;307:1613–23. 36869203 10.1007/s00404-023-06967-0

[39] Stadler JT Scharnagl H Wadsack C Marsche G. Preeclampsia affects lipid metabolism and HDL function in mothers and their offspring. Antioxidants (Basel). 2023;12:795. 37107170 10.3390/antiox12040795 PMC10135112

[40] Sánchez-Aranguren LC Prada CE Riaño-Medina CE Lopez M. Endothelial dysfunction and preeclampsia: role of oxidative stress. Front Physiol. 2014;5:372. 25346691 10.3389/fphys.2014.00372 PMC4193194

[41] Redman CW Sargent IL. Latest advances in understanding preeclampsia. Science. 2005;308:1592–4. 15947178 10.1126/science.1111726

[42] Spracklen CN Smith CJ Saftlas AF Robinson JG Ryckman KK. Maternal hyperlipidemia and the risk of preeclampsia: a meta-analysis. Am J Epidemiol. 2014;180:346–58. 24989239 10.1093/aje/kwu145 PMC4565654

[43] Maierean SM Mikhailidis DP Toth PP. The potential role of statins in preeclampsia and dyslipidemia during gestation: a narrative review. Expert Opin Investig Drugs. 2018;27:427–35. 10.1080/13543784.2018.1465927 29672173

[44] Nakimuli A Starling JE Nakubulwa S. Relative impact of pre-eclampsia on birth weight in a low resource setting: a prospective cohort study. Pregnancy Hypertens. 2020;21:1–6. 32330863 10.1016/j.preghy.2020.04.002 PMC7450268

[45] Iijima T Obata S Miyagi E Aoki S. Clinical features of preeclampsia preceded by fetal growth restriction. Cureus. 2023;15:e51275. 38288232 10.7759/cureus.51275 PMC10823203

[46] Chappell LC Brocklehurst P Green ME. Planned early delivery or expectant management for late preterm pre-eclampsia (PHOENIX): a randomised controlled trial. Lancet. 2019;394:1181–90. 31472930 10.1016/S0140-6736(19)31963-4 PMC6892281

[47] Rana S Powe CE Salahuddin S. Angiogenic factors and the risk of adverse outcomes in women with suspected preeclampsia. Circulation. 2012;125:911–9. 22261192 10.1161/CIRCULATIONAHA.111.054361 PMC3319742

[48] Rana S Hacker MR Modest AM. Circulating angiogenic factors and risk of adverse maternal and perinatal outcomes in twin pregnancies with suspected preeclampsia. Hypertension. 2012;60:451–8. 22753210 10.1161/HYPERTENSIONAHA.112.195065 PMC3432569

[49] Reddy M Palmer K Rolnik DL Wallace EM Mol BW Da Silva Costa F. Role of placental, fetal and maternal cardiovascular markers in predicting adverse outcome in women with suspected or confirmed pre-eclampsia. Ultrasound Obstet Gynecol. 2022;59:596–605. 34985800 10.1002/uog.24851

[50] Verlohren S Herraiz I Lapaire O. The sFlt-1/PlGF ratio in different types of hypertensive pregnancy disorders and its prognostic potential in preeclamptic patients. Am J Obstet Gynecol. 2012;206:58.e1–8. 10.1016/j.ajog.2011.07.037 22000672

