# Supplementary material for: Predictors of adverse pregnancy outcomes in severe preeclampsia: A retrospective observational study
Source: Medicine (Baltimore). 2025 Apr 25;104(17):e42258. doi: 10.1097/MD.0000000000042258 (PMC12040025; doi:10.1097/MD.0000000000042258)
Supplement: Supplementary file 1 [file medi-104-e42258-s001.docx]

**Supplemental Digital Content Table 1 Medication Use for Hypertension Management Between Groups**

| **Medication Use** | **Control group (n=167)** | **Adverse outcome group (n=184)** | **χ²** | **P-value** |
| --- | --- | --- | --- | --- |
| Magnesium sulfate | 165 (98.80%) | 183 (99.46%) | 0.442 | 0.506 |
| Nifedipine | 163 (97.60%) | 182 (98.91%) | 0.892 | 0.345 |
| Labetalol | 167 (100%) | 184 (100%) | 0.000 | 1.000 |
