# Supplementary material for: Predictors of adverse pregnancy outcomes in severe preeclampsia: A retrospective observational study
Source: Medicine (Baltimore). 2025 Apr 25;104(17):e42258. doi: 10.1097/MD.0000000000042258 (PMC12040025; doi:10.1097/MD.0000000000042258)
Supplement: Supplementary file 2 [file medi-104-e42258-s002.docx]

**Supplemental Digital Content Table 2. Univariate Logistic Regression Analysis**

| **Variables** | **OR (95% CI)** | **P-value** |
| --- | --- | --- |
| Gestational age at delivery (weeks) | 0.494 (0.422-0.579) | <0.001 |
| Twin pregnancy | 5.088 (2.606-9.936) | <0.001 |
| IVF-ET use | 1.947 (1.174-3.228) | 0.010 |
| ICU admission | 3.889 (2.249-6.725) | <0.001 |
|  |  |  |
| PlGF | 0.998 (0.997-0.999) | 0.001 |
| Total cholesterol | 1.210 (1.043-1.403) | 0.012 |
| Triglycerides | 1.106 (0.991-1.234) | 0.071 |
| Maternal age | 1.017 (0.976-1.060) | 0.427 |
| BMI | 0.985 (0.933-1.041) | 0.593 |
| GDM | 0.892 (0.567-1.403) | 0.621 |
| HBV infection | 1.009 (0.400-2.547) | 0.985 |

BMI: Body mass index; GDM: Gestational diabetes mellitus; HBV: Hepatitis B virus; ICU: Intensive care unit; IVF-ET: In vitro fertilization-embryo transfer; PlGF: Placental growth factor
