# Supplementary material for: Predictors of adverse pregnancy outcomes in severe preeclampsia: A retrospective observational study
Source: Medicine (Baltimore). 2025 Apr 25;104(17):e42258. doi: 10.1097/MD.0000000000042258 (PMC12040025; doi:10.1097/MD.0000000000042258)
Supplement: Supplementary file 3 [file medi-104-e42258-s003.docx]

**Supplemental Digital Content Table 3. Diagnostic value of gestational age, placental growth factor, and total cholesterol for adverse pregnancy outcomes**

| **Variables** | **Cutoff value** | **AUC** | **95% CI** | **Sensitivity (%)** | **Specificity (%)** |
| --- | --- | --- | --- | --- | --- |
| Gestational age | 36.5 | 0.858 | 0.816-0.901 | 79.89 | 92.22 |
| PlGF | 118.29 | 0.685 | 0.629-0.740 | 59.78 | 71.86 |
| Total cholesterol | 6.805 | 0.573 | 0.513-0.633 | 44.02 | 70.06 |
| Gestational age + PlGF + total cholesterol | 0.506 | 0.867 | 0.826-0.908 | 79.35 | 90.42 |

AUC: area under the curve; CI: confidence interval; PlGF: placental growth factor.
